# The relationship between consciousness and the ascending reticular activating system in patients with traumatic brain injury

**DOI:** 10.1186/s12883-020-01942-7

**Published:** 2020-10-14

**Authors:** Sung Ho Jang, Young Hyeon Kwon

**Affiliations:** Department of Physical Medicine and Rehabilitation, College of Medicine, Yeungnam University 317-1, Daemyungdong, Namku, Taegu, 705-717 Republic of Korea

**Keywords:** Consciousness, Ascending reticular activating system, Traumatic brain injury, Diffusion tensor tractography, Glasgow coma scale

## Abstract

**Background:**

We investigated the relationship between consciousness and the ascending reticular activating system (ARAS) by using diffusion tensor tractography (DTT) in patients with traumatic brain injury (TBI).

**Methods:**

Twenty-six patients with TBI and 13 healthy control subjects were recruited for this study. Glasgow Coma Scale (GCS) scores were used for evaluation of subject consciousness state at the chronic stage of TBI (at DTT scanning), According to the GCS score, the patient group was divided into two subgroups: A (14 patients;impaired consciousness: GCS score < 15, and B (12 patients;intact consciousness;GCS score = 15). Fractional anisotropy (FA) and tract volume (TV) values were assessed in the lower dorsal and upper ARAS.

**Results:**

The FA values of the lower dorsal ARAS and the upper ARAS in patient subgroup A were significantly lower than those in patient subgroup B and the control group(*p* <  0.05). However, the FA and TV values for the lower dorsal ARAS and the upper ARAS were not significantly different between patient subgroup B and the control group(*p* > 0.05). The FA value of the lower dorsal ARAS(*r* = 0.473,*p* <  0.05) and the TV of upper ARAS(*r* = 0.484,*p* <  0.05) had moderate positive correlations with the GCS score. The FA value of the upper ARAS had a strong positive correlation with the GCS score of the patient group(*r* = 0.780,*p* <  0.05).

**Conclusions:**

We detected a close relationship between consciousness at the chronic stage of TBI and injuries of the lower dorsal and upper ARAS (especially, the upper ARAS) in patients who showed impaired consciousness at the onset of TBI. We believe that our results can be useful during the development of therapeutic strategies for patients with impaired consciousness following TBI.

**Trial registration:**

YUMC 2019–06–032-003. Retrospectively registered 06 Jun 2020.

## Background

Traumatic brain injury (TBI) is a major cause of neurological disability in adults [1, 2]. Impaired consciousness is a common and serious sequela following TBI, and it has been reported that approximately half of patients in a vegetative state at 1 month after TBI remain in the vegetative state until 1 year after onset [1, 2]. Therefore, research on the relation between consciousness and the neural structures that are related to consciousness in TBI is important in terms of the development of appropriate therapeutic strategies and for prognosis prediction. With regard to this field of research, little is known although a few studies have reported that there are relationships between consciousness and various neural structures including the corpus callosum, internal capsule, thalamus, and brainstem in patients with TBI [3–5].

Consciousness, which consists of arousal and awareness of oneself and the environment, is not fully understood, but it is known to be controlled by a complicated series of complex actions involving various neural structures [6–10]. The ascending reticular activating system (ARAS) has been considered as a main neural structure for consciousness [10–12]. The ARAS is a complicated network that connects a portion of the brainstem reticular formation (RF) with nonspecific thalamic nuclei, the basal forebrain, hypothalamus, and the cerebral cortex [10–12].

Before the development of diffusion tensor imaging (DTI), precise evaluation of the ARAS in the human brain was limited because the majority of the ARAS is not clearly discriminated from adjacent neural structures. By contrast, diffusion tensor tractography (DTT), which is derived from DTI data, enables three-dimensional reconstruction, visualization, and evaluation of the ARAS in the live human brain [12–14]. A few studies have demonstrated the usefulness of DTT for three-dimensional evaluation of the ARAS in patients with impaired consciousness following TBI [12, 15, 16]. However, very little has been reported about the relationship between consciousness and the ARAS in TBI.

In this study, we hypothesized that consciousness, as assessed by the Glasgow Coma Scale (GCS) score, would be related with DTT results obtained for the ARAS in TBI patients [17]. Thus, we investigated the relationship between GCS and the ARAS in patients with TBI, using DTT.

## Methods

### Subjects

A total of 26 consecutive patients with TBI (mean age 48.2 years, range 20–71 years) and 13 age- and sex-matched normal control subjects (mean age 42.7 years, range 29–63 years) with no history of TBI or neurologic/psychiatric disease were recruited into this study. Inclusion criteria for the 26 patients were as follow: (1) first ever TBI; (2) impaired consciousness (GCS score < 15) at the onset of TBI; (3) DTI scanning at more than 1 month after the onset of TBI; (4) age at the time of head trauma, 20–75 years; (5) no previous history of TBI or neurologic/psychiatric disease. This study was performed retrospectively, and the study protocol was approved by the institutional review board of our university hospital.

### Clinical evaluation

The GCS is a representative and validated scale for describing consciousness in TBI and is used for evaluation of the conscious state [17]. The GCS consists of three components (eye-opening, verbal, motor) and the total GCS score ranges between 3 and 15 [17]. The GCS score of the study patients was acquired twice (at TBI onset and during the chronic stage) DTI scanning was performed during the chronic stage. We classified the patient group (26 patients) into two subgroups based on their GCS score at the time of DTI scanning: 14 patients were placed in subgroup A (impaired consciousness; GCS score < 15), and 12 patients were assigned to subgroup B (intact consciousness; GCS score = 15). No significant difference in GCS scores at TBI onset was detected between subgroups A and B (Table 1). However, GCS score at DTI scanning was significantly higher in subgroup B than in subgroup A (*p* <  0.05).

**Table 1:** Demographic and clinical data of the patient and control groups

	Patient group (*n* = 26)		Control group (*n* = 13)
	subgroup A (*n* = 14)	subgroup B (*n* = 12)	
Mean age, years	43.78 (±20.38)	51.8 (±11.8)	42.7 (±10.02)
GCS score			
At onset	4.86 (±2.1)	6.3 (±3.7)	–
At DTI scanning^*^	9.71 (±1.73)	15 (±0.0)	–
Mean duration to DTI (months)	5.2 (±5.0)	3.6 (±4.3)	–

Values represent mean (±standard deviation); *GCS* Glasgow Coma Scale, *DTI* Diffusion tensor imaging

^*^Significantly different between patient subgroups A and B at *p* < 0.05

### Diffusion tensor imaging and fiber tracking

**Fig. 1:**
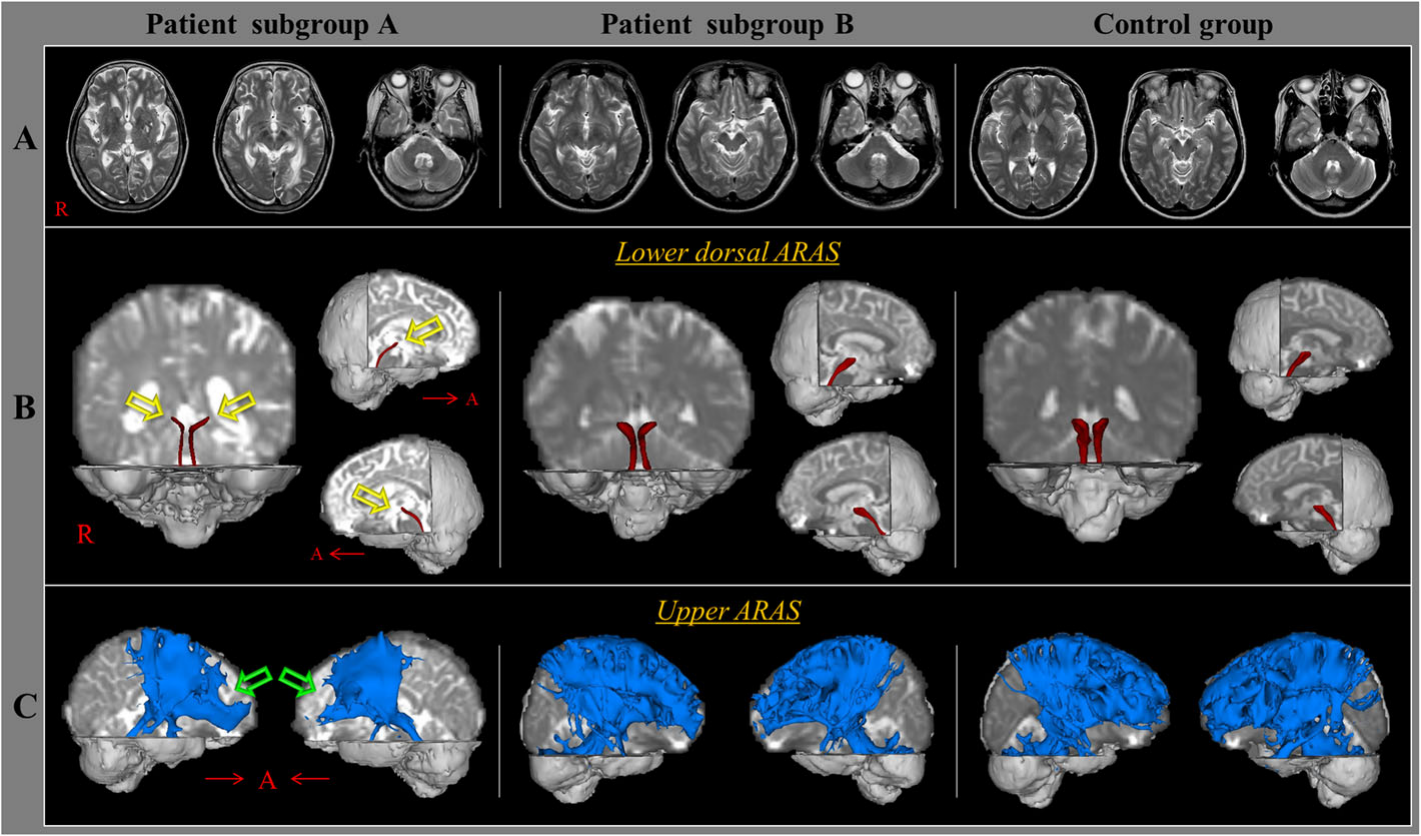
Results of diffusion tensor tractography (DTT) for the ascending reticular activating system (ARAS). **a** T2-weighted brain magnetic resonance images at the time of diffusion tensor imaging scanning in representative subjects of patient subgroup A (71-year-old female), patient subgroup B (33-year-old male), and the control group (50-year-old female). **b** Results of DTT for the lower dorsal ARAS. Narrowing (yellow arrows) is observed in both lower dorsal ARAS in patient subgroup A compared to that in patient subgroup B and the control group. **c** Results of DTT for the upper ARAS. A decreased neural tract (green arrows) is observed in both upper ARAS in patient subgroup A compared with that in patient subgroup B and the control group

Acquisition of DTI data was performed at an average of 4.7 ± 4.4 months after TBI onset by using a 6-channel head coil on a 1.5 T Philips Gyroscan Intera (Philips, Best, Netherlands) and single-shot echo-planar imaging. For each of the 32 noncollinear diffusion sensitizing gradients, 67 contiguous slices were acquired parallel to the anterior commissure–posterior commissure line. Imaging parameters were as follows: acquisition matrix = 96 × 96, reconstructed to matrix = 192 × 192 matrix, field of view = 240 mm × 240 mm, TR = 10,398 ms, TE = 72 ms, parallel imaging reduction factor (SENSE factor) = 2, EPI factor = 59 and *b* = 1000 s/mm^2^, NEX = 1, slice gap = 0, and slice thickness = 2.5 mm. Fiber tracking was performed by applying a probabilistic tractography method based on a multifiber model using tractography routines implemented in the FMRIB Diffusion Toolbox (https://fsl.fmrib.ox.ac.uk/fsl/fslwiki/FDT). Two portions of the ARAS were reconstructed by selecting fibers passing through the following regions of interest (ROIs): the lower dorsal ARAS (seed ROI: the pontine RF; target ROI: the thalamic intralaminar nucleus (ILN)), and the upper ARAS (seed ROI: ILN; target ROI: cerebral cortex) [13, 14]. Of the 5000 samples generated from the seed voxel, results for positive contacts were visualized at a minimum threshold of 2 for the lower dorsal ARAS. For reconstruction of the neural connectivity of the ILN a threshold of 10 contacts streamlined through each voxel were used for analysis. Values of fractional anisotropy (FA) and tract volume (TV) of the two portions of the ARAS assessed in this study were determined (Fig. 1).

### Statistical analysis

Statistical analysis was performed by using the SPSS 12.0 for Windows (SPSS, Chicago, IL, USA). An independent t-test was used for determination of the significance of the variances in GCS scores between subgroups A and B. One-way analysis of variance (ANOVA) with Fisher’s least significant difference *post-hoc* test was performed to determine the significance of differences in DTT parameters (FA and TV) of the ARAS between patient subgroups A and B and the control group. In addition, Pearson correlation coefficients were used to assess the correlations between FA and TV values of patient subgroups A and B and their GCS scores. Results were considered significant when the *p* value was < 0.05. A correlation coefficient of more than 0.60 indicated a strong correlation, a correlation coefficient between 0.40 and 0.59 indicated a moderate correlation, while that between 0.20 and 0.39 indicated a weak correlation, and one less than 0.19 indicated a very weak correlation [18].

## Results

Comparisons of the FA and TV DTI parameters for lower dorsal and upper ARAS of patient subgroups A and B and the control group are summarized in Table 2. One-way ANOVA showed that no significant differences were observed in the mean TV values of the lower dorsal and upper ARAS among patient subgroups A, B and the control group (*p* > 0.05). However, the mean FA values of the lower dorsal and upper ARAS were significantly different among patient subgroup A, B and control group (F = 7.10, *p* <  0.05 and F = 39.16, *p* < 0.05). Post hoc tests revealed that the mean FA values of the lower dorsal and the upper ARAS in patient subgroup A were significantly different from those of patient subgroup B and the control group groups (*p* < 0.05) (Table 2).

**Table 2:** Diffusion tensor tractography parameters of lower dorsal and upper ARAS of the subgroup A, B and control groups

	Subgroup A	Subgroup B	Control group	F	P
Lower dorsal ARAS					
FA	0.68 (±0.12) ^a^	0.77 (±0.06) ^b^	0.81 (±0.02) ^b^	7.10	< 0.05*
TV	730.84 (±425.17)	950.08 (±412.12)	953.61 (±156.14)	0.69	0.20
Upper ARAS					
FA	0.60 (±0.05)^a^	0.69 (±0.04)^b^	0.72 (±0.03)^b^	39.16	< 0.05*
TV	38,751.28 (±16,162.42)	47,132.42 (±9651.04)	47,267.00 (±7883.63)^b^	2.23	0.12

Values represent mean (±standard deviation); *ARAS* Ascending reticular activating system, *FA* Fractional anisotropy, *TV* Tract volume; One-way ANOVA and the LSD post hoc test were used for comparison of diffusion tensor parameters

*significant difference between the patient and control groups at the indicated p

LSD test result: a < b

The correlations between the FA and TV DTT parameters and the GCS score at the time of DTI scanning of the TBI patient group are shown in Table 3. The FA value of the lower dorsal ARAS (*r* = 0.473, *p* < 0.05) and the TV of upper ARAS (*r* = 0.484, *p* < 0.05) had moderate positive correlations with the GCS score at DTI scanning of the patient group [18]. The FA value of the upper ARAS had a strong positive correlation with the GCS score of the patient group (*r* = 0.780, *p* < 0.05). By contrast, the TV of the lower dorsal ARAS in the patient group had a weak correlation with the GCS score of the patient group (*r* = 0.243, *p* > 0.05).

**Table 3:** Correlation between Glasgow Coma Scale score and diffusion tensor tractography parameters in the patient group

	Lower dorsal ARAS		Upper ARAS	
	FA	TV	FA	TV
r	0.473	–	0.780	0.484
*p* value	< 0.05^*^	–	< 0.05^*^	< 0.05^*^

Values represent mean (±standard deviation); *ARAS* Ascending reticular activating system, *FA* Fractional anisotropy, *TV* Tract volume, *r* Correlation coefficient

^*^correlation is significant at *p* < 0.05

## Discussion

In this study, the relationship between GCS score during the chronic stage of TBI and the DTT results for the ARAS was investigated in patients who showed impaired consciousness at TBI onset. We observed the following: first, the FA values of the lower dorsal ARAS and the upper ARAS in patient subgroup A were lower than those of patient subgroup B and the control group; second, the FA value of the lower dorsal ARAS and the TV of the upper ARAS had moderate positive correlations with the GCS score obtained during the chronic stage of TBI in the patient group. Furthermore, the FA value of the upper ARAS had a strong positive correlation with the GCS score during the chronic stage in the patient group.

Among the DTT parameters, FA and TV are most commonly used in evaluating the status of neural tracts in patients with brain injury [19, 20]. The FA value represents the state of white matter organization by indicating the degree of directionality and integrity of white matter microstructures such as axons, myelin, and microtubules, with a low FA value suggesting a loss of white matter integrity [19]. The TV value is determined by the number of voxels included in a neural tract, thereby suggesting the total number of fibers within the tract [20]. Therefore, low FA and/or TV values for a neural tract indicate an injury of that neural tract [19, 20]. As a result, in this study, the low FA values of the lower dorsal ARAS and upper ARAS in patient subgroup A (patients exhibited impaired consciousness during the chronic stage of TBI) indicate injuries of these neural tracts.

Regarding the correlation between GCS score and DTT parameters at the chronic stage in the patient group, we observed that the FA value of the lower dorsal ARAS and the TV of the upper ARAS had moderate positive correlations with GCS score, and the FA value of the upper ARAS had a strong positive correlation with the GCS score. These results suggest that the injury severities of the lower dorsal and upper ARAS were related to the level of consciousness in the chronic stage of TBI. In particular, compared to the lower dorsal ARAS, the upper ARAS was more closely associated with the level of consciousness [18].

Since the introduction of DTI, a few studies have demonstrated that the injuries of several neural structures such as the corpus callosum, internal capsule, thalamus, and brainstem were related to consciousness in patients with TBI [3–5]. A few case reports have demonstrated ARAS injuries in patients with impaired consciousness following TBI [12, 15, 16]. In 2013, Edlow et al. reported on a patient with coma following a severe TBI who showed complete disruption of white matter pathways connecting the brainstem arousal nuclei to the basal forebrain and thalamic nuclei, as well as partial disruption of the pathways connecting the thalamus and basal forebrain to the cerebral cortex [12]. Subsequently, Jang et al. (2015) demonstrated recovery of an injured lower dorsal ARAS concurrent with impaired consciousness in a patient with severe TBI [15]. In 2016, Jang et al. demonstrated the recovery of an injured upper ARAS with concomitant recovery of impaired consciousness in a patient with TBI and hypoxic-ischemic brain injury [16]. As a result, to the best of our knowledge, the present DTT-based study is the first to demonstrate a relationship between consciousness and the ARAS in a large number of patients with TBI. However, some limitations of this study need to be considered. First, brain regions with fiber complexity and crossing can prevent the full DTI visualization of the underlying fiber architecture; therefore, DTI may underestimate or overestimate the state of some fiber tracts [21]. Second, this retrospective study included a relatively small number of subjects. Third, because this study was performed retrospectively, we were not able to obtain the other neuropsychological data, except for GCS. Thus, prospective studies that include a larger number of subjects should be encouraged.

## Conclusion

In conclusion, we found a close relationship between consciousness at the chronic stage of TBI and injuries of the lower dorsal and upper ARAS (especially, the upper ARAS) in patients who showed impaired consciousness at the onset of TBI. We believe that our observation can be useful in the development of therapeutic strategies for patients with impaired consciousness following TBI.

## Data Availability

The datasets generated and/or analyzed during the current study are not publicly available due to institutional restrictions but are available from the corresponding author on reasonable request.

## Abbreviations

TBI: Traumatic brain injury
ARAS: Ascending reticular activating system
RF: Reticular formation
DTI: Diffusion tensor imaging
DTT: Diffusion tensor tractography
GCS: Glasgow coma scale
ROIs: Regions of interest
ILN: Intralaminar nucleus
FA: Fractional anisotropy
TV: Tract volume

## References

[1] Godbolt AK, Deboussard CN, Stenberg M, Lindgren M, Ulfarsson T, Borg J (2013). Disorders of consciousness after severe traumatic brain injury: a swedish-icelandic study of incidence, outcomes and implications for optimizing care pathways. J Rehabil Med.

[2] Giacino JT, Ashwal S, Childs N, Cranford R, Jennett B, Katz DI (2002). The minimally conscious state: definition and diagnostic criteria. Neurology.

[3] Huisman TA, Schwamm LH, Schaefer PW, Koroshetz WJ, Shetty-Alva N, Ozsunar Y (2004). Diffusion tensor imaging as potential biomarker of white matter injury in diffuse axonal injury. AJNR Am J Neuroradiol.

[4] Rutgers DR, Fillard P, Paradot G, Tadie M, Lasjaunias P, Ducreux D (2008). Diffusion tensor imaging characteristics of the corpus callosum in mild, moderate, and severe traumatic brain injury. AJNR Am J Neuroradiol.

[5] Haberg AK, Olsen A, Moen KG, Schirmer-Mikalsen K, Visser E, Finnanger TG (2015). White matter microstructure in chronic moderate-to-severe traumatic brain injury: impact of acute-phase injury-related variables and associations with outcome measures. J Neurosci Res.

[6] Paus T (2000). Functional anatomy of arousal and attention systems in the human brain. Prog Brain Res.

[7] Moruzzi G, Magoun HW (1949). Brain stem reticular formation and activation of the eeg. Electroencephalogr Clin Neurophysiol.

[8] Laureys S, Schiff ND (2012). Coma and consciousness: paradigms (re) framed by neuroimaging. Neuroimage.

[9] Majerus S, Gill-Thwaites H, Andrews K, Laureys S (2005). Behavioral evaluation of consciousness in severe brain damage. Prog Brain Res.

[10] Zeman A (2001). Consciousness. Brain.

[11] Gosseries O, Bruno MA, Chatelle C, Vanhaudenhuyse A, Schnakers C, Soddu A (2011). Disorders of consciousness: what's in a name?. NeuroRehabilitation.

[12] Edlow BL, Takahashi E, Wu O, Benner T, Dai G, Bu L (2012). Neuroanatomic connectivity of the human ascending arousal system critical to consciousness and its disorders. J Neuropathol Exp Neurol.

[13] Yeo SS, Chang PH, Jang SH (2013). The ascending reticular activating system from pontine reticular formation to the thalamus in the human brain. Front Hum Neurosci.

[14] Jang SH, Lim HW, Yeo SS (2014). The neural connectivity of the intralaminar thalamic nuclei in the human brain: a diffusion tensor tractography study. Neurosci Lett.

[15] Jang SH, Kim SH, Lim HW, Yeo SS (2015). Recovery of injured lower portion of the ascending reticular activating system in a patient with traumatic brain injury. Am J Phys Med Rehabil.

[16] Jang S, Kim S, Lee H (2016). Recovery from vegetative state to minimally conscious state: a case report. Am J Phys Med Rehabil.

[17] Teasdale G, Maas A, Lecky F, Manley G, Stocchetti N, Murray G (2014). The Glasgow coma scale at 40 years: standing the test of time. Lancet Neurol.

[18] Cohen J (1988). Statistical power analysis for the behavioral sciences.

[19] Mori S, Crain BJ, Chacko VP, van Zijl PC (1999). Three-dimensional tracking of axonal projections in the brain by magnetic resonance imaging. Ann Neurol.

[20] Assaf Y, Pasternak O (2008). Diffusion tensor imaging (DTI)-based white matter mapping in brain research: a review. J Mol Neurosci.

[21] Yamada K, Sakai K, Akazawa K, Yuen S, Nishimura T (2009). MR tractography: a review of its clinical applications. Magn Reson Med Sci.

